# Mn/carbon aerogels derived from the water-induced self-assembly of UIO-66 (Mn) for the thermal decomposition of ammonium perchlorate

**DOI:** 10.3389/fchem.2024.1427451

**Published:** 2024-06-17

**Authors:** Jun Zhao, Yang Liu, Hui Wang, Xu cheng Fu, Neng mei Deng

**Affiliations:** ^1^ Key Laboratory of Biomimetic Sensor and Detecting Technology of Anhui Province, School of Materials and Chemical Engineering, West Anhui University, Lu’an, China; ^2^ Sichuan College of Architectural Technology, Deyang, China; ^3^ The 2nd Geological Brigade of Sichuan, Chengdu, China

**Keywords:** UIO-66 (Mn), Mn/C aerogels, AP, catalytic performance, kinetics

## Abstract

In solid propellants, combustion catalysts play a crucial role. Here, we introduce a convenient method for the self-assembly of UIO-66 (Mn) in the presence of water, leading to the preparation of Mn/C aerogels. The aerogels were successfully utilized in the thermocatalytic decomposition of ammonium perchlorate (AP). The results indicate that the incorporation of 2% mass fraction of Mn/C aerogels enhances the peak temperature of AP decomposition by approximately 87.5°C. Mn/C aerogels demonstrate excellent catalytic performance. In combination with kinetics, we propose a thermal catalytic mechanism.

## 1 Introduction

Ammonium perchlorate (AP) is the most prevalent oxidizer used in solid fuel rocket composite propellants, comprising 60%–90% of the total propellant mass. The combustion performance of composite propellants, particularly their burning rate, is significantly influenced by the reaction rate, activation energy, and pyrolysis temperature of AP (Lufei et al., 2024; Songnan et al., 2024). Adding combustion catalysts is an effective way to improve the burning rate of propellants as it can reduce the peak decomposition temperature and activation energy of AP, thus enhancing the combustion performance of AP-based propellants. Commonly used catalysts include elemental metals and metal oxides, which typically have low catalytic efficiency and are prone to agglomeration (Nafise Modanlou and Tarighi, 2020; Dan et al., 2021).

Researchers in the field of modifying traditional metal catalysts have conducted extensive research in order to achieve higher stability and improved catalytic efficiency. Metal–organic framework (MOFs) materials possess highly controllable porous structures and extremely high specific surface areas, making them have broad application potential in areas such as gas adsorption, separation and storage, and catalyst supports (Juan et al., 2022; Tao et al., 2022; Juan et al., 2024). The structures and compositions can be precisely adjusted according to specific needs, endowing them with multifunctionality and sustainability (Sanoop et al., 2017). So, MOFs showcase vast prospects for applications in the field of catalysis due to their controllability, porosity, high surface area, and multifunctionality. Based on the formation of coordination bonds between metal ions and organic ligands, MOFs have been extensively studied as ideal sacrificial templates for various applications in the synthesis of carbon-based nanomaterials (Chao et al., 2014; Jitendra Kumar et al., 2016). The resulting carbon-based nanomaterials possess advantages such as large specific surface area, high thermal stability, and layered porous structures, exhibiting various excellent performances (Jingjing et al., 2022).

**FIGURE 1:**
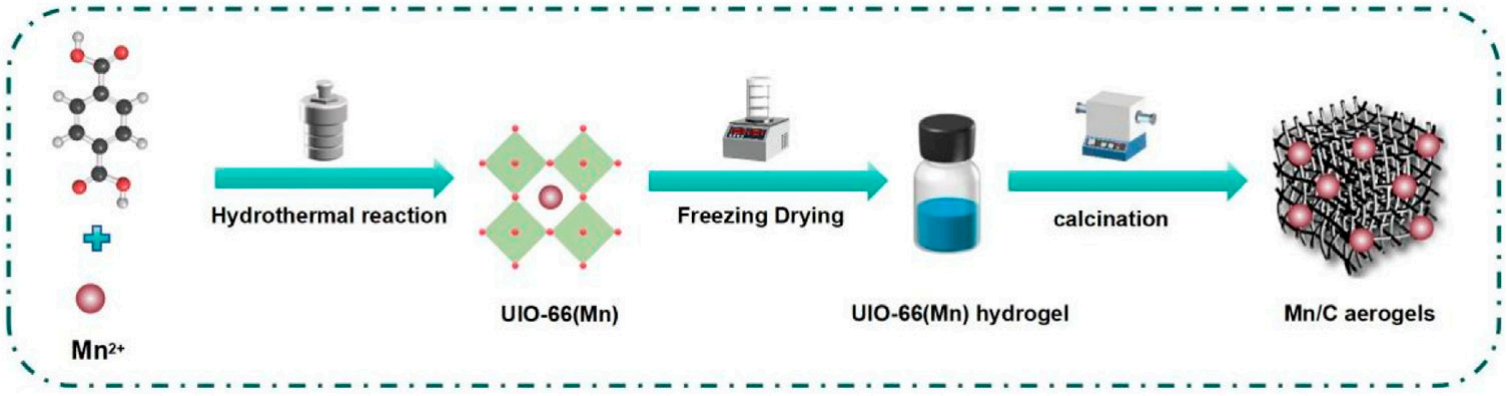
Schematic illustration of the preparation process of Mn/C aerogels.

In particular, Mn can form multiple valence states, and the change in its valence state will also change its ion radius and ion potential. Mn nanoparticles or single atoms often play a crucial role in a series of chemical energy conversion processes as they possess various physical and chemical properties due to their electronegativity, which can better adapt to different needs. As shown in Figure 1, we reported a simple strategy for the synthesis of Mn/carbon (Mn/C) aerogels by the water-induced self-assembly of Mn-based metal–organic frameworks (UIO-66 (Mn)). The classic UIO-66 (Mn) with well-defined structures was prepared via a one-pot method. Subsequently, the UIO-66 (Mn) hydrogel was obtained through freeze-drying, followed by calcination to obtain the Mn/C aerogels.

## 2 Experimental section

### 2.1 Materials

Manganese(II) chloride (MnCl_2_) (≥99.9%), 1,4-dicarboxybenzene (PTA, ≥99.9%), and ethyl alcohol (75%) were purchased from Sinopharm Chemical Reagent Co., Ltd., and used without modification.

### 2.2 Preparation of UIO-66 (Mn)

UIO-66 (Mn) was prepared using the hydrothermal method (Farrando-Pérez et al., 2023). Typically, a calculated amount of MnCl_2_ was mixed with PTA at a molar ratio of 1:1. MnCl_2_ (125.84 mg, 1 mmol) and PTA (166.13 mg, 1 mmol) were dissolved in 10 mL of ethyl alcohol and continued to be transferred to a reactor. The oven temperature was set at 120°C, and the reaction lasted for 12 h. The mixture was naturally cooled to room temperature to obtain purple crystal particles. After filtration, the target product was washed three times with ethanol.

### 2.3 Preparation of ultralight Mn/C aerogels

Second, 220 mg of UIO-66 (Mn) was mixed with 6 mL ethanol and 54 mL deionized water under ultrasonication for 5 min. The color of the mixture changed from pink to light purple. After standing for 24 h, UIO-66 (Mn)-assembled hydrogel was formed. Subsequently, the freeze-dried UIO-66 (Mn)-assembled hydrogel was heated to 800°C under a nitrogen atmosphere at a heating rate of 5°C/min in the tube furnace. Finally, keeping the same heating rate to higher temperatures (800°C) for 1 h, the as-proposed Mn/C aerogels were achieved after cooling down to room temperature.

### 2.4 Measurement of catalytic properties

The thermal decomposition performances of all the synthesized samples were evaluated using differential scanning calorimetry (DSC), which was implemented over a range of 50°C–600°C at heating rates of 10, 15, 20, and 25 °C/min under an argon flow (50 mL/min).

### 2.5 Crystal structures

The single crystal of UIO-66 (Mn) was mounted on a glass fiber randomly. The single-crystal X-ray diffraction data on UIO-66 (Mn) were collected using a Bruker Smart APEX II CCD diffractometer equipped with graphite-monochromatic Mo-Kα radiation (=0.71073 Å) via an ω scan mode in the range of 2.17°< θ < 27.94° at 100.01(10) K. The collected diffraction points included independent diffraction points. The structure was solved by direct methods with the SHELXS-97 program.

## 3 Results and discussion

### 3.1 Vibrational modes and structural analysis

**FIGURE 2:**
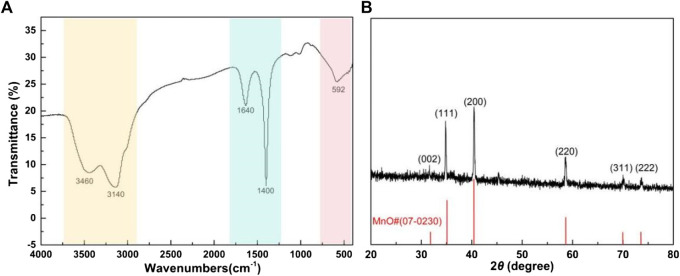
FT-IR **(A)** and XRD **(B)** of Mn/C aerogels.

The FT-IR is shown in Figure 2A. The absorption at 592 cm^−1^ was the Mn-C stretching vibration peak, and the two peaks at 3,460 cm^−1^ and 3,140 cm^−1^ were caused by the stretching vibration of -OH and C-H bonds, respectively (Huang et al., 2023). The peak at 1,640 cm^−1^ was generated from the bending O-H vibration (Zhang Y. et al., 2023a), and the absorption peak at 1,400 cm^−1^ was caused by the C=C vibration. Figure 2B shows the XRD of the Mn/C aerogel catalyst. The peak at 32° corresponded to the diffraction peak of carbon (002), and the five diffraction peaks observed at 35, 40, 58, 70, and 75° corresponded to the (111), (200), (220), (311), and (222) crystal plane diffraction peaks of manganese(II), respectively. The diffraction peak showed that manganese mainly existed in the form of MnO in the Mn/C aerogels, and the crystallinity was good (Zhu et al., 2016; Zhu et al., 2018). The calculation of diffraction powder grain size was mainly based on the FWHM, and the calculated result was approximately 80 nm.

**FIGURE 3:**
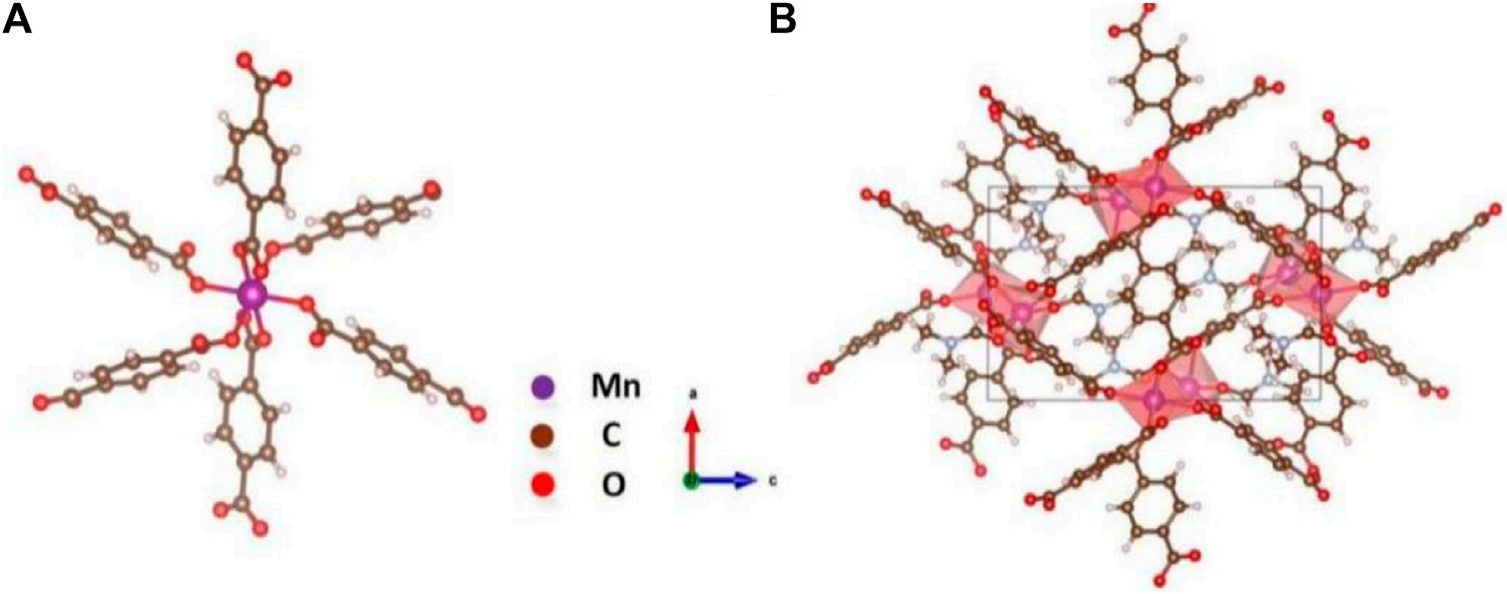
**(A)** Unit lattice of UIO-66 (Mn) and **(B)** crystal structure of UIO-66 (Mn).

The unit lattice and crystal structure of the sample are shown in Figure 3A. The unit lattice and crystal structure of UIO-66 (Mn) were confirmed by single-crystal diffraction measurements. First, nanoparticles were dissolved in methanol. The methanol solution was slowly evaporated at room temperature to obtain purple crystals. The single-crystal size was 0.2 mm × 0.15 mm × 0.1 mm. The data on UIO-66 (Mn) are summarized in Table 1. The structure was solved by the direct method using the SHELXS-97 program. As a result, the formula was C_36_H_40_Mn_3_N_4_O_16_. During the test, 25,600 reflection points were collected, revealing that UIO-66 (Mn) belongs to the triclinic crystal system, with the P-1 space group, a density of 1.589 g/cm^3^, and a molecular weight of 949.54. Figure 3B shows that manganese(II) functions as the coordinating metal center, which coordinates with six terephthalic acids to form a stable Mn-O-C structure.

**TABLE 1:** Crystal data and structure refinement for UIO-66 (Mn).

CCDC	2352727
Empirical formula	C_36_H_40_Mn_3_N_4_O_16_
Formula weight	949.54
Temperature/K	100.01(10)
Crystal system	Triclinic
Space group	P-1
a/Å	9.8812(3)
b/Å	12.5447(6)
c/Å	16.5960(7)
α/°	74.955(4)
β/°	88.989(3)
γ/°	87.397(3)
Volume/Å^3^	1,984.58(14)
Z	2
ρ_calc_g/cm^3^	1.589
μ/mm^−1^	1.018
F(000)	974.0
Crystal size/mm^3^	0.2 × 0.15 × 0.1
Radiation	Mo Kα (λ = 0.71073)
2Θ range for data collection/°	3.364 to 52.744
Index ranges	−12 ≤ h ≤ 11, −15 ≤ k ≤ 15, and −20 ≤ l ≤ 20
Reflections collected	25,600
Independent reflections	8,015 [R_int_ = 0.0476, R_sigma_ = 0.0453]
Data/restraints/parameters	8,015/0/540
Goodness-of-fit on F^2^	1.066
Final R indexes [I ≥ 2σ (I)]	R_1_ = 0.0631, wR_2_ = 0.1698
Final R indexes [all data]	R_1_ = 0.0800, wR_2_ = 0.1821
Largest diffraction peak/hole/e Å^-3^	1.64/-0.66

### 3.2 Textural properties and morphology

**FIGURE 4:**
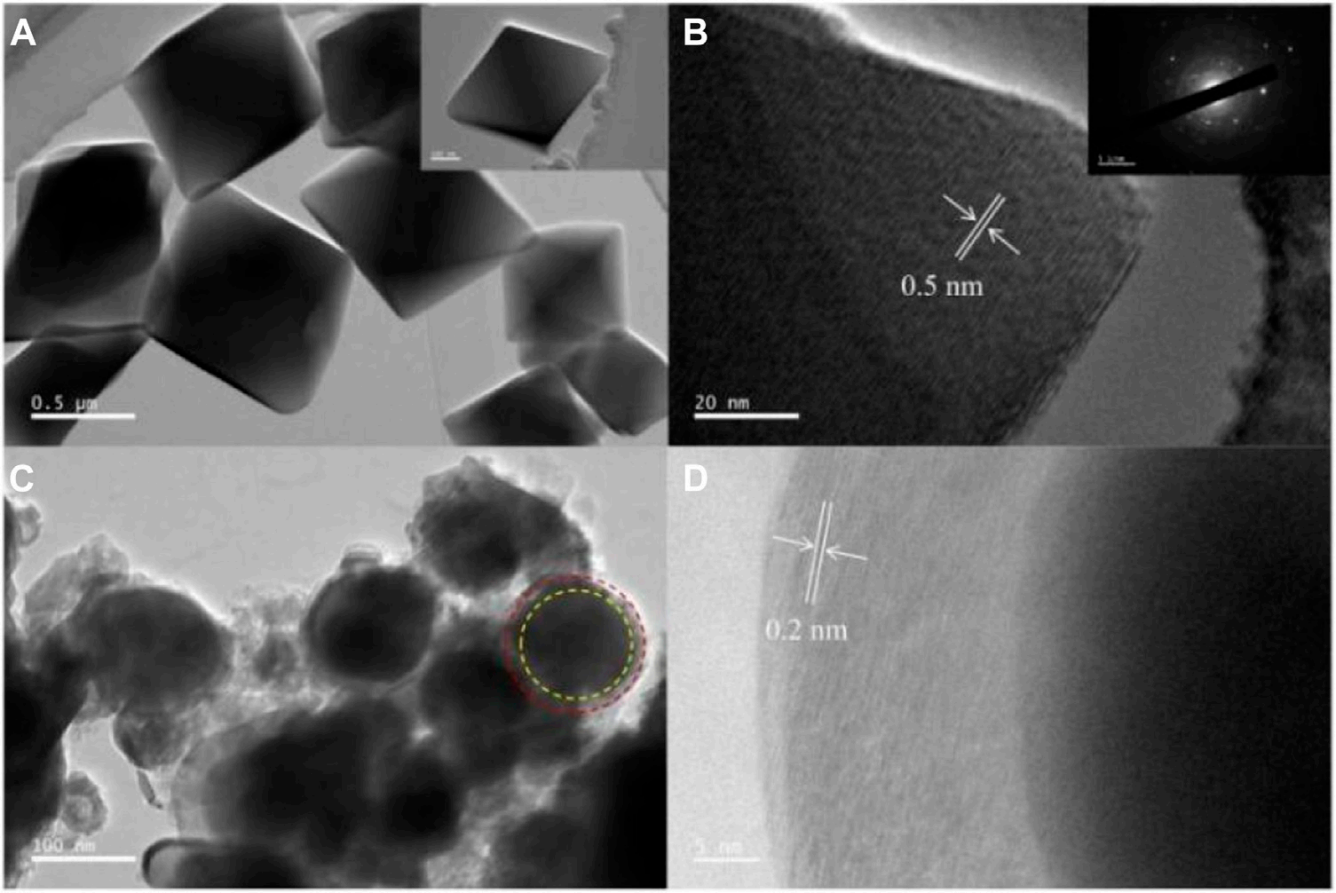
TEM **(A)** and HR-TEM **(B)** images of UIO-66 (Mn), and TEM **(C)** and HR-TEM **(D)** images of Mn/C aerogels.

Figure 4A shows the TEM image of UIO-66 (Mn). We can clearly observe that UIO-66 (Mn) crystal particles exhibited a regular octahedral morphology at the microscopic level, and their distribution was very uniform. The one-dimensional size of a single particle was approximately 0.5 mm. Figure 4B shows the resulting images of HRTEM tests conducted on individual UIO-66 (Mn) particles, with a lattice stripe spacing of 0.5 nm and diffraction patterns resembling typical polycrystalline patterns. Figure 4C shows the manganese/carbon aerogel calcined at high temperature (Daneshmand-Jahromi et al., 2022). By comparing with the morphology of UIO-66 (Mn), the target product presented a spherical core–shell structure. The outer layer was a two-dimensional carbon structure formed after the carbonization of organic ligands in UIO-66 (Mn), and the inner layer was a metal center, presenting a uniform and stable structure. Similarly, we conducted HRTEM tests on it (Figure 4D), and the lattice fringes were 0.2 nm.

**FIGURE 5:**
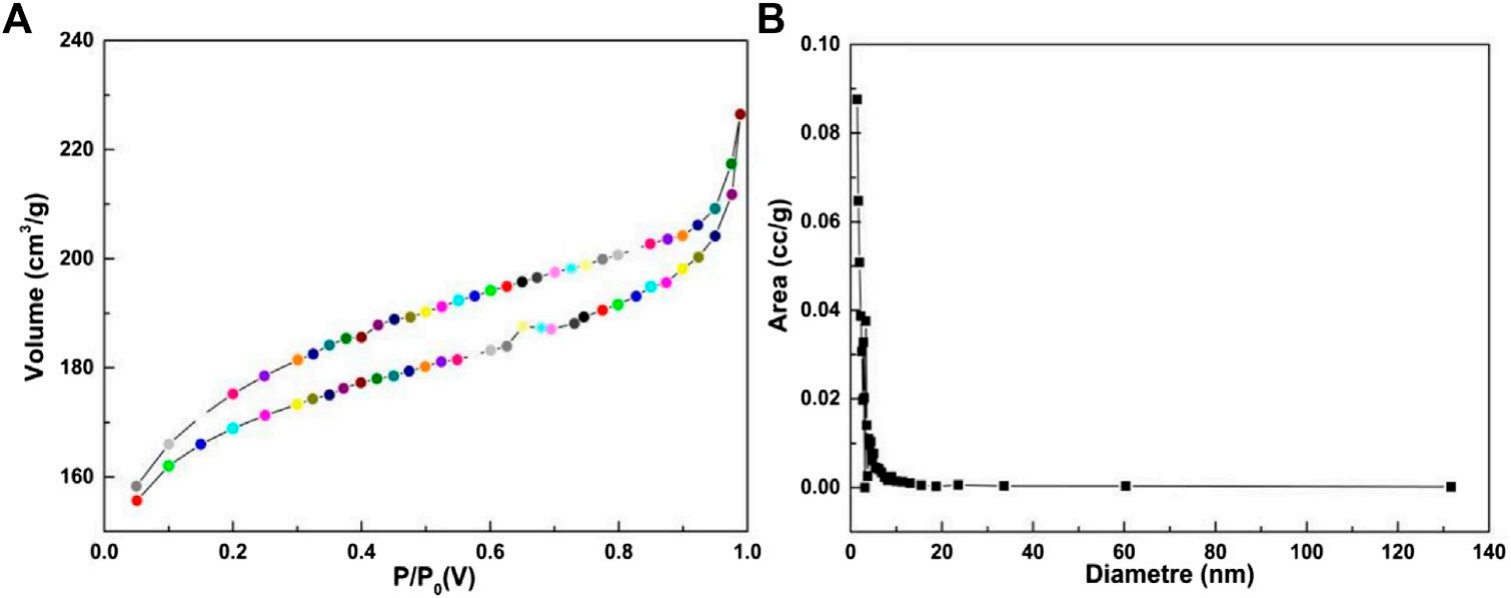
N_2_ adsorption/desorption isotherms **(A)** and the pore size distribution **(B)** of Mn/C aerogels.

As shown in Figure 5A, the nitrogen adsorption isotherm test was used to evaluate the porosity of the catalyst. In Figure 5B, the Mn/C aerogels exhibited typical reversible adsorption, indicating that the interior of the material was mainly mesoporous. The BET model was used to calculate the surface area of the material to be 521.939 m^2^g^−1^. The pore size was mainly distributed at approximately 10 nm and had a small pore structure. The large surface area of Mn/C aerogels was due to the strong hydrogen bonding generated by the inner manganese particles, which ensured structural stability and improved the flatness of the 3D layer. These will help improve the structural order of aerogels and increase the active surface area (Wu et al., 2023).

### 3.3 Thermal behavior

**FIGURE 6:**
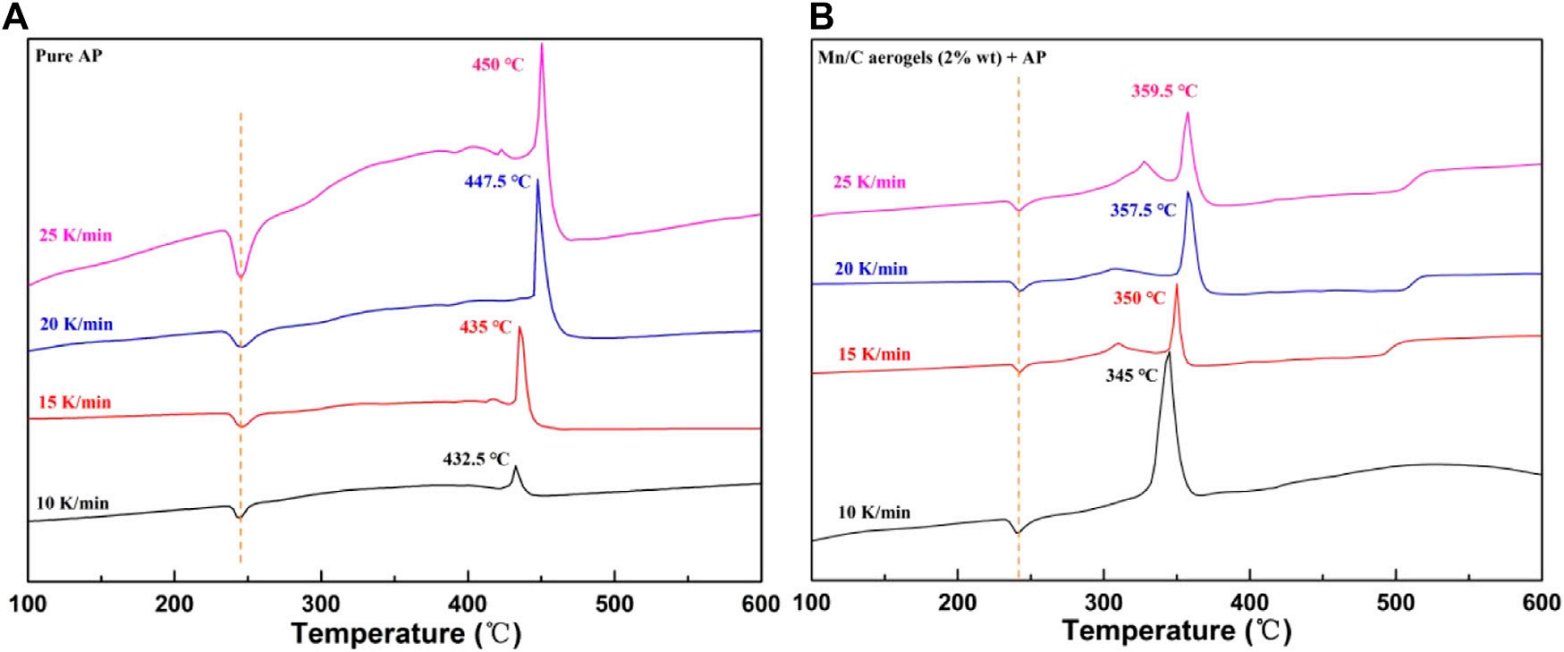
DSC curves of pure AP **(A)** and AP with 2% wt Mn/C aerogels **(B)** at 10, 15, 20, and 25 K/min.

As is well known, AP thermal decomposition can be divided into two stages: low temperature and high temperature. The first stage was at approximately 320°C, and the high temperature decomposition was at approximately 420°C (Song et al., 2024). As shown in Figure 6, we analyzed the catalytic effect of Mn/C aerogels on AP from the perspective of thermal decomposition kinetics and calculated the thermal decomposition activation energy of pure AP and the thermal decomposition activation energy after adding Mn/C aerogels using the Kissinger equation (Dong et al., 2022; Yan et al., 2022). Figures 6A, B represent the DSC curves of pure AP and AP with 2% wt Mn/C aerogels at different heating rates, respectively. It was evident that when Mn/C aerogels were added, the peak temperature of AP high-temperature decomposition was advanced by approximately 87.5°C.

We have studied the thermal catalytic process from the perspectives of thermal decomposition kinetics. The relationship between the AP decomposition temperature and the rate of warming can be expressed according to the Kissinger equation, Eq. 1, where β is the heating rate, K·min^−1^; T_*P*_ is the peak temperature; R is the ideal gas constant, 8.314 J mol·K^−1^; A is the finger front factor; and E_a_ is the activation energy, J·mol^−1^ (Tan et al., 2015; Hu et al., 2020).

$$\ln\left(\frac{\beta }{T_{P}^{2}}\right)=\ln\left(\frac{AR}{E_{a}}\right)-\frac{E_{a}}{RT_{P}}.$$ (1)

**FIGURE 7:**
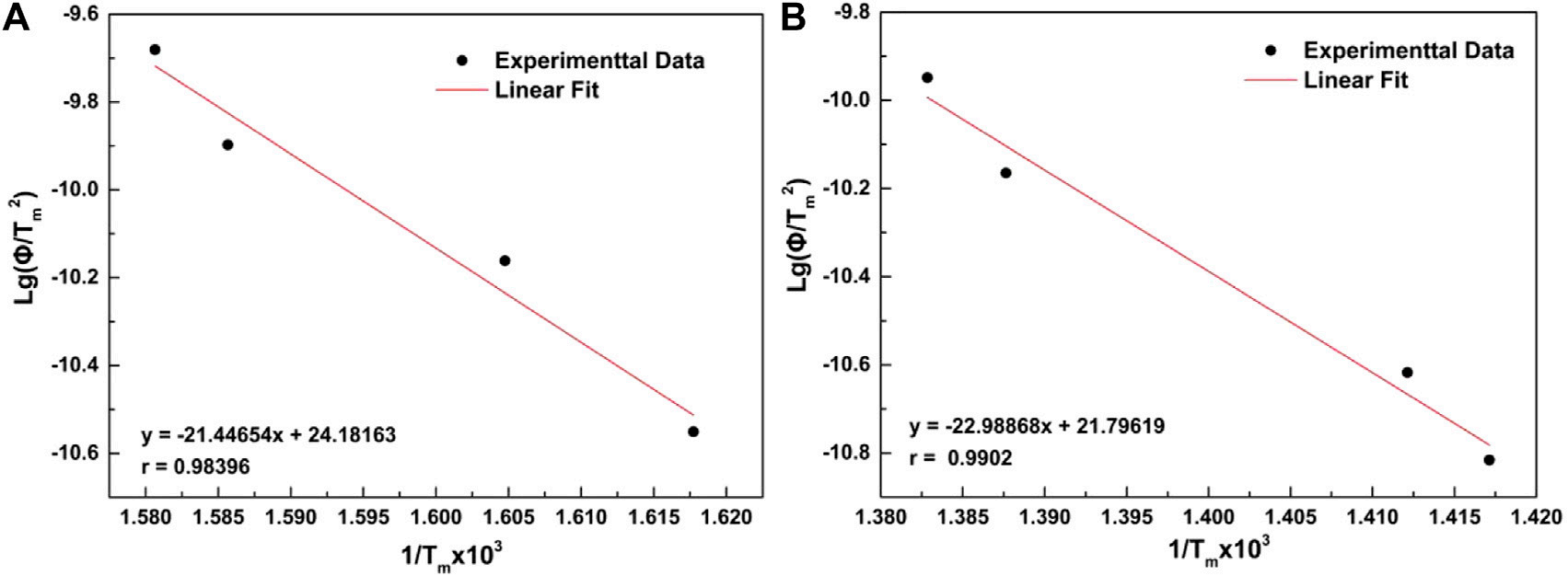
Fitting curve of pure AP **(A)** and AP with 2% wt Mn/C aerogels **(B)**.

The calculated kinetic parameters of the AP and AP with Mn/C aerogels are shown in Tables 2, 3. According to the kinetic experimental data, ln(β/ $T_{P}^{2}$ was plotted against 1000/ $T_{P}$ to obtain the fitting curve so that the activation energy could be calculated, and the E_a_ of pure AP was 190.85 kJ mol^−1^, which is shown in Figure 7A; however, as shown in Figure 7B, when Mn/C aerogels were added, E_a_ decreased to 178.30 kJ mol^−1^. Based on the DSC curves, the catalyst mainly acts on the high-temperature decomposition stage of AP, and the addition of Mn/C aerogels changes the thermal decomposition process of AP. (Garci et al., 2015; Pei et al., 2021).

**TABLE 2:** Calculated kinetic parameters of AP.

Φ (K/min)	T_m_ (K)	1/T_m_×10^3^	ln(Φ/T_m_^2^)	lgΦ
10	705.65	1.417	−10.815	1
15	708.15	1.412	−10.417	1.176
20	720.65	1.387	−10.164	1.301
25	723.15	1.382	−9.948	1.397

**TABLE 3:** Calculated kinetic parameters of AP with Mn/C aerogels.

Φ (K/min)	T_m_ (K)	1/T_m_×10^3^	ln(Φ/T_m_^2^)	lgΦ
10	618.15	1.617	−10.550	1
15	623.15	1.604	−10.161	1.176
20	630.65	1.585	−9.897	1.301
25	632.65	1.580	−9.680	1.397

**FIGURE 8:**
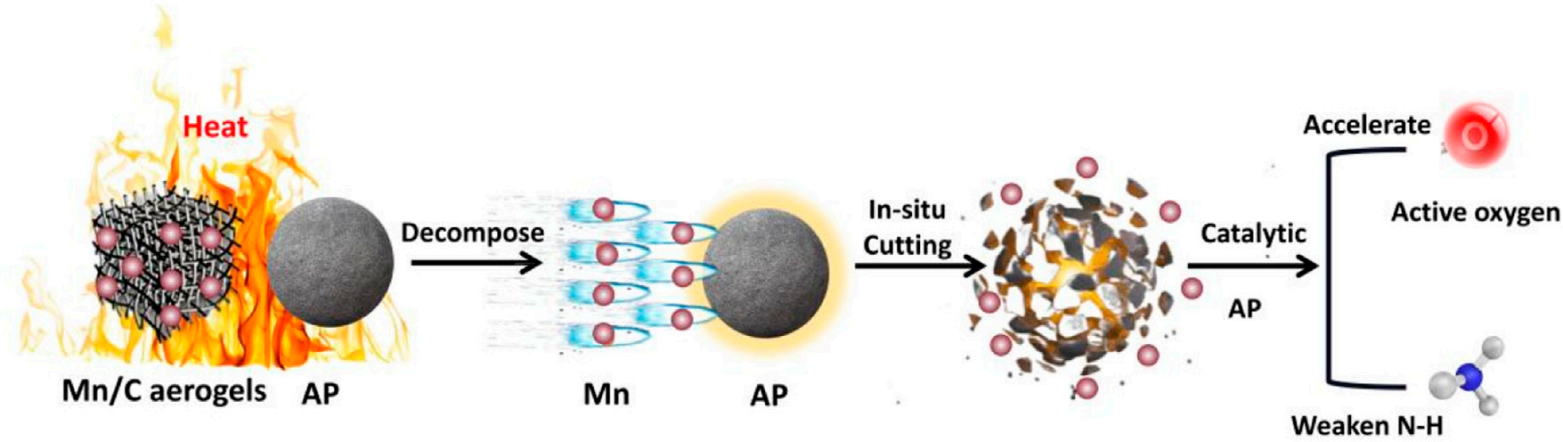
Thermal decomposition mechanism of AP.

According to the thermal decomposition kinetic test results of AP and our previous research on the thermal catalysis of AP, such as TiO_2_ NPs/h-BN: preparation and catalytic activities of a novel AP catalyst (Frontiers in Chemistry 10 (2022): 947052), it can be concluded that the catalytic mechanism of Mn/C aerogels on AP can be divided into four processes, namely, adsorption, activation, targeted catalysis, and desorption, as shown in Figure 8 (Zhang F. et al., 2023). (Ⅰ) Adsorption: AP is adsorbed on the surface of carbon aerogels. At the same time, the intermolecular van der Waals force further improves the bond cooperation to improve the reaction activity. (Ⅱ) Activation: Under high-temperature conditions, Mn rapidly impacts AP particles, cutting AP *in situ* and increasing surface energy. Then, AP can undergo redox reactions with active sites on the surface of Mn, forming reactions NH_3_^*^ and H^*^. (Ⅲ) Targeted catalysis: The active sites on the surface of Mn can provide an effective reaction site, allowing the chemical transformation of ammonium perchlorate molecules at this site. This process inhibits the decomposition of N-H and promotes the formation of **·**O_2_^−^. (Ⅳ) Desorption: After the catalytic reaction is complete, the product is desorbed from the surface of the Mn and released. Mn can be used again to adsorb other ammonium perchlorate molecules for the next round of catalytic cycle.

## 4 Conclusion

The results showed that we successfully used a convenient strategy for the water-induced self-assembly of UIO-66 (Mn) to prepare Mn/C aerogels. It was found that Mn/C aerogels can be used as an efficient thermal catalyst for AP. Adding 2% wt to AP advanced the decomposition peak temperature of AP by approximately 87.5°C and reduced the thermal decomposition activation energy of AP by approximately 12.55 kJ mol^−1^. Furthermore, the kinetics theoretical calculation showed that the active site on the surface of Mn provided an effective reaction site, allowing the chemical conversion of AP molecules at this site. This process inhibited the decomposition of N-H and promoted the formation of ·O_2_^−^. Based on this, we propose a four-step mechanism for the thermal decomposition of AP.

## Data Availability

The data presented in the study are deposited in the Cambridge Structural Database, accession number 2352727.
