# Supplementary material for: Mn/carbon aerogels derived from the water-induced self-assembly of UIO-66 (Mn) for the thermal decomposition of ammonium perchlorate
Source: Front Chem. 2024 Jun 17;12:1427451. doi: 10.3389/fchem.2024.1427451 (PMC11215826; doi:10.3389/fchem.2024.1427451)

checkCIF/PLATON report

Structure factors have been supplied for datablock(s) 6258-1_auto

THIS REPORT IS FOR GUIDANCE ONLY. IF USED AS PART OF A REVIEW PROCEDURE FOR PUBLICATION, IT SHOULD NOT REPLACE THE EXPERTISE OF AN EXPERIENCED CRYSTALLOGRAPHIC REFEREE.

No syntax errors found. CIF dictionary Interpreting this report

Datablock: 6258-1_auto

```
Bond precision:   C-C = 0.0070 A          Wavelength=0.71073

Cell:          a=9.8812(3)       b=12.5447(6)      c=16.5960(7)
               alpha=74.955(4)   beta=88.989(3)    gamma=87.397(3)
Temperature:   100 K
```

	Calculated	Reported
Volume	1984.58(14)	1984.58(14)
Space group	P -1	P -1
Hall group	-P 1	-P 1
Moiety formula	C36 H40 Mn3 N4 O16 [+ solvent]	C36 H40 Mn3 N4 O16
Sum formula	C36 H40 Mn3 N4 O16 [+ solvent]	C36 H40 Mn3 N4 O16
Mr	949.54	949.54
Dx,g cm-3	1.589	1.589
Z	2	2
Mu (mm-1)	1.018	1.018
F000	974.0	974.0
F000’	976.49	
h,k,lmax	12,15,20	12,15,20
Nref	8129	8015
Tmin,Tmax	0.833,0.903	0.770,1.000
Tmin’	0.816	

```
Correction method= # Reported T Limits: Tmin=0.770 Tmax=1.000
AbsCorr = MULTI-SCAN

Data completeness= 0.986            Theta(max)= 26.372
```

R(reflections)= 0.0631(6373)

wR2(reflections)= 0.1821(8015)

S = 1.066   Npar= 540

The following ALERTS were generated. Each ALERT has the format
test-name_ALERT_alert-type_alert-level.
Click on the hyperlinks for more details of the test.

Alert level C

PLAT094_ALERT_2_C Ratio of Maximum / Minimum Residual Density ....   2.47 Report
PLAT341_ALERT_3_C Low Bond Precision on C-C Bonds ....   0.007 Ang.
PLAT411_ALERT_2_C Short Inter H...H Contact H31   ..H31   .   2.14 Ang.
2-x,1-y,1-z =   2_766 Check
PLAT911_ALERT_3_C Missing FCF Refl Between Thmin & STh/L=   0.600   77 Report
5 1 0,   7 1 0, -5 2 0,   1 3 0,   8 10 0,   6 -2 1,
-5 -1 1, -7 0 2,   6 0 2,   8 0 2,   3 1 2, -4 -2 3,
3 -1 3,   4 -1 3, -6 0 3, -4 0 3, -2 0 3,   5 0 3,
7 0 3,   9 0 3, 11 3 3,   7 -1 4, -3 0 4,   1-11 5,
5 -2 5, -5 -1 5,   6 -1 5, -8 1 5, -6 1 5,   3 -2 6,
6 -2 6, -4 -1 6,   8 -1 6,   3 0 6,   9 0 6, -3 -1 7,
0 -1 7,   1 -1 7,   2 -1 7, -5 0 7,   8 11 7, -7 -1 8,
2 -1 8,   4 -1 8,   7 0 8, -6 -1 9,   9 -1 9,   0 -9 10,
-3 -3 11, -7 -2 11,   1 13 11,   2 0 13,   8 7 13,   3 -2 14,
7 7 14,   7 8 14,   2 -6 15,   2 -5 15,   2 -5 16,   2 -4 16,
6 8 16,   2 -3 17,   2 2 17,   2 3 17,   2 4 17,   2 -2 18,
2 -1 18,   2 0 18,   2 3 18,   2 4 18,   2 7 18,   2 1 19,
3 3 19,   2 4 19,   3 4 19,   2 5 19,   3 5 19,
PLAT971_ALERT_2_C Check Calcd Resid. Dens. 1.07Ang From Mn3   1.60 eA-3
PLAT971_ALERT_2_C Check Calcd Resid. Dens. 0.95Ang From Mn2   1.54 eA-3
PLAT971_ALERT_2_C Check Calcd Resid. Dens. 1.03Ang From Mn2   1.53 eA-3

Alert level G

PLAT004_ALERT_5_G Polymeric Structure Found with Maximum Dimension   2 Info
PLAT083_ALERT_2_G SHELXL Second Parameter in WGHT Unusually Large   10.26 Why ?
PLAT605_ALERT_4_G Largest Solvent Accessible VOID in the Structure   7 A**3
PLAT794_ALERT_5_G Tentative Bond Valency for Mn1   (II)   .   2.19 Info
PLAT794_ALERT_5_G Tentative Bond Valency for Mn2   (II)   .   2.03 Info
PLAT794_ALERT_5_G Tentative Bond Valency for Mn3   (II)   .   2.23 Info
PLAT794_ALERT_5_G Tentative Bond Valency for Mn4   (II)   .   2.01 Info
PLAT868_ALERT_4_G ALERTS Due to the Use of _smtbx_masks Suppressed   ! Info
PLAT910_ALERT_3_G Missing # of FCF Reflection(s) Below Theta(Min).   1 Note
0 0 1,
PLAT912_ALERT_4_G Missing # of FCF Reflections Above STh/L= 0.600   36 Note
PLAT930_ALERT_2_G FCF-based   Twin Law (1 0 0)   Est.d BASF   0.15 Check
PLAT931_ALERT_5_G CIFcalcFCF Twin Law (1 0 0)   Est.d BASF   0.15 Check
PLAT933_ALERT_2_G Number of HKL-OMIT Records in Embedded .res File   48 Note
-8 1 5, -7 -2 11, -7 -1 8, -7 0 2, -6 -1 9, -6 0 3,
-6 1 5, -5 -1 1, -5 -1 5, -5 0 7, -5 2 0, -4 -2 3,
-4 -1 6, -4 0 3, -3 -3 11, -3 -1 7, -3 0 4, -2 0 3,
0 -1 7,   1 -1 7,   1 3 0,   1 13 11,   1 15 7,   2 -1 7,
2 -1 8,   3 -2 6,   3 -2 14,   3 -1 3,   3 0 6,   3 1 2,
4 -1 3,   4 -1 8,   5 -2 5,   5 0 3,   5 1 0,   6 -2 1,
6 -2 6,   6 -1 5,   6 0 2,   7 -1 4,   7 0 3,   7 0 8,

```
              7  1  0,   8 -1  6,   8  0  2,   9 -1  9,   9  0  3,   9  0  6,
PLAT941_ALERT_3_G Average HKL Measurement Multiplicity ..........        3.2 Low
PLAT969_ALERT_5_G The 'Henn et al.' R-Factor-gap value ..........       4.31 Note
              Predicted wR2: Based on SigI**2  4.22 or SHELX Weight 17.68
PLAT978_ALERT_2_G Number C-C Bonds with Positive Residual Density.         0 Info
```

```
   0 ALERT level A = Most likely a serious problem - resolve or explain
   0 ALERT level B = A potentially serious problem, consider carefully
   7 ALERT level C = Check. Ensure it is not caused by an omission or oversight
  16 ALERT level G = General information/check it is not something unexpected

   0 ALERT type 1 CIF construction/syntax error, inconsistent or missing data
   9 ALERT type 2 Indicator that the structure model may be wrong or deficient
   4 ALERT type 3 Indicator that the structure quality may be low
   3 ALERT type 4 Improvement, methodology, query or suggestion
   7 ALERT type 5 Informative message, check
```

It is advisable to attempt to resolve as many as possible of the alerts in all categories. Often the minor alerts point to easily fixed oversights, errors and omissions in your CIF or refinement strategy, so attention to these fine details can be worthwhile. In order to resolve some of the more serious problems it may be necessary to carry out additional measurements or structure refinements. However, the purpose of your study may justify the reported deviations and the more serious of these should normally be commented upon in the discussion or experimental section of a paper or in the "special_details" fields of the CIF. checkCIF was carefully designed to identify outliers and unusual parameters, but every test has its limitations and alerts that are not important in a particular case may appear. Conversely, the absence of alerts does not guarantee there are no aspects of the results needing attention. It is up to the individual to critically assess their own results and, if necessary, seek expert advice.

Publication of your CIF in IUCr journals

A basic structural check has been run on your CIF. These basic checks will be run on all CIFs submitted for publication in IUCr journals (*Acta Crystallographica*, *Journal of Applied Crystallography*, *Journal of Synchrotron Radiation*); however, if you intend to submit to *Acta Crystallographica Section C* or *E* or *IUCrData*, you should make sure that full publication checks are run on the final version of your CIF prior to submission.

Publication of your CIF in other journals

Please refer to the *Notes for Authors* of the relevant journal for any special instructions relating to CIF submission.

PLATON version of 06/01/2024; check.def file version of 05/01/2024

Datablock 6258-1_auto - ellipsoid plot

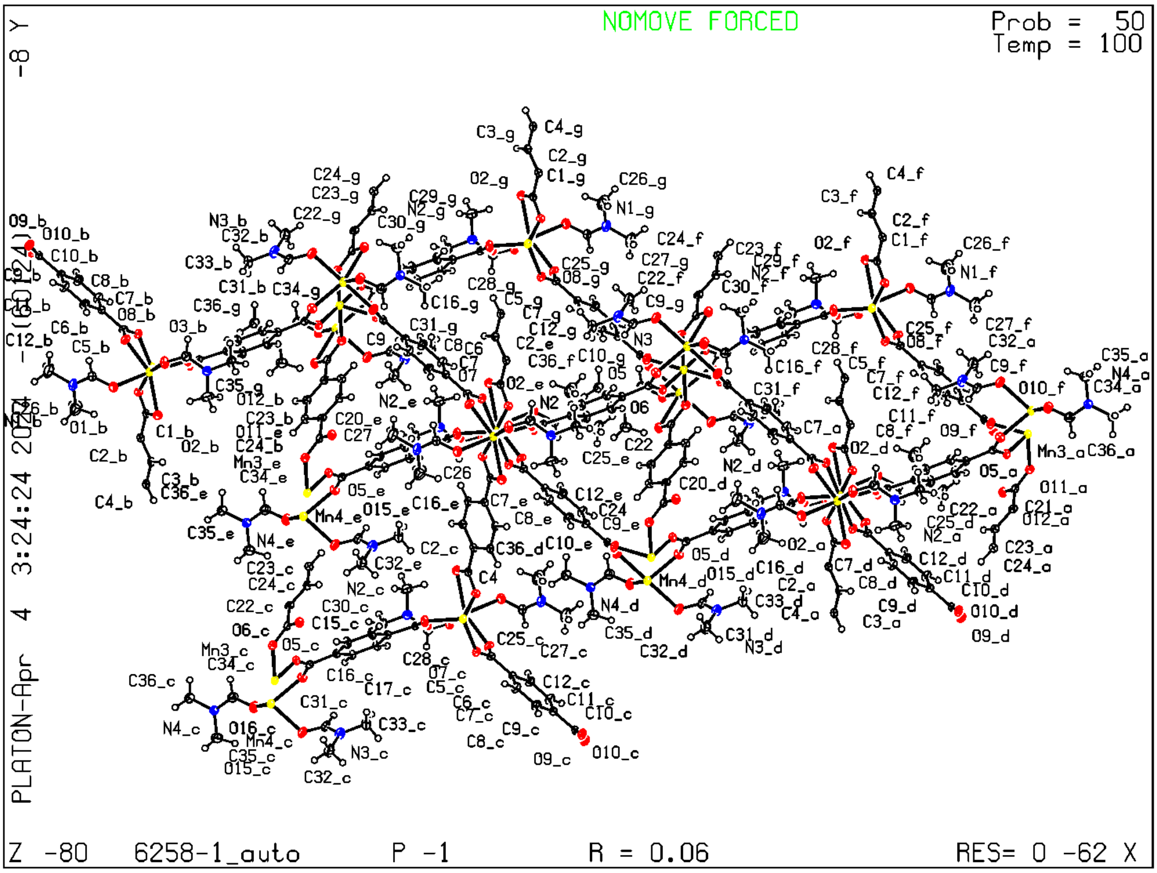

Supplement: Supplementary file 1 [file DataSheet1.PDF]
